# Ordering structured populations in multiplayer cooperation games

**DOI:** 10.1098/rsif.2015.0881

**Published:** 2016-01

**Authors:** Jorge Peña, Bin Wu, Arne Traulsen

**Affiliations:** Department of Evolutionary Theory, Max Planck Institute for Evolutionary Biology, August-Thienemann-Straße 2, Plön 24306, Germany

**Keywords:** evolutionary game theory, spatial structure, order theory, computational geometry, stochastic orders

## Abstract

Spatial structure greatly affects the evolution of cooperation. While in two-player games the condition for cooperation to evolve depends on a single structure coefficient, in multiplayer games the condition might depend on several structure coefficients, making it difficult to compare different population structures. We propose a solution to this issue by introducing two simple ways of ordering population structures: the containment order and the volume order. If population structure 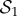 is greater than population structure 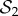 in the containment or the volume order, then 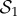 can be considered a stronger promoter of cooperation. We provide conditions for establishing the containment order, give general results on the volume order, and illustrate our theory by comparing different models of spatial games and associated update rules. Our results hold for a large class of population structures and can be easily applied to specific cases once the structure coefficients have been calculated or estimated.

## 1. Introduction

The evolution of cooperation is a fascinating topic that has been studied from different perspectives and theoretical approaches [1–5]. An issue that has led to considerable interest is the extent to which spatial structure allows cooperation to thrive [6–28]. Spatial structure can both enhance cooperation by inducing clustering or assortment (whereby cooperators tend to interact more often with other cooperators [11,29,30]) and oppose cooperation by inducing increased local competition (whereby cooperators tend to compete more often with other cooperators [31]). For two-player games or multiplayer games with similar strategies, the balance between these two opposing effects is captured by the ‘scaled relatedness coefficient’ of inclusive fitness theory [15,19,27,28] or the ‘structure coefficient’ of evolutionary game theory [6,16,22]. These coefficients are functions of demographic parameters, and take into account the degree of assortment, the effects of density dependence and the strength of local competition resulting from spatial interactions [10,15,22]. Two different models of spatial structure and associated evolutionary dynamics can be unambiguously compared by ranking their relatedness or structure coefficients: the greater the coefficient, the less stringent the conditions for cooperation to evolve. Hence, different models of population structure can be ordered by their potential to promote the evolution of cooperation in a straightforward way.

Despite the theoretical importance of models leading to a single relatedness or structure coefficient, many examples of social evolution ranging from microbial cooperation [32–34] to collective action in humans [35–37] involve games between more than two players with distinct strategies [38,39]. In these cases, the effects of spatial structure cannot be captured by a single coefficient, as higher degrees of association (e.g. ‘triplet relatedness’ [17,40]) are required to fully describe the condition under which cooperation is favoured [27,41,42]. The need to account for several structure coefficients has so far precluded a simple way of comparing population structures independently of the particular game used to model cooperation.

**Figure 1.**
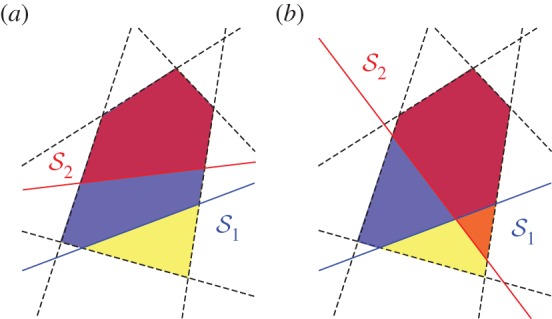
Containment and volume orders of cooperation. The set of *d*-player cooperation games is defined by a set of linear inequalities (dashed lines) defining a polytope in a 2*d*-dimensional space. A given population structure (e.g. 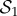 or 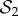) is characterized by a selection condition defining a further linear inequality (solid lines). Here, we show a pictorial representation of the projection of such multidimensional objects to the plane, where polytopes are polygons. (*a*) The set of games for which cooperation is favoured under 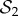 is contained in the set of the games for which cooperation is favoured under 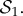 Hence, we say that 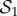 is greater than 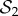 in the containment order (and write 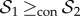). (*b*) 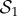 and 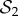 cannot be ordered in the containment order as there are both games for which 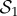 favours cooperation but not 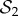 (purple polygon), and games for which 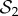 favours cooperation but not 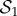 (orange polygon). In both panels, 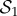 favours cooperation for more games than 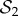 does. Hence, we say that 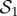 is greater than 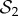 in the volume order (and write 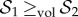).

Here, we propose a framework to order population structures by their potential to promote cooperation that is also valid in the case of games between multiple players with distinct strategies. Our framework allows the comparison of two population structures without referring to any concrete game. We will distinguish two cases, depending on the inclusion relation between the sets of games for which cooperation is promoted under each population structure. (i) The set of games for which the second population structure promotes cooperation is fully contained in the set of games for which the first population structure promotes cooperation (figure 1*a*). In this case, we say that the first population structure is greater than the second in the containment order, and hence a stronger promoter of cooperation. (ii) The set of games for which one population structure promotes cooperation is not fully contained in the set of games for which the other population structure promotes cooperation (figure 1*b*). In this case, we say that the population structure promoting cooperation for a larger volume of games is greater in the volume order, and hence a stronger promoter of cooperation.

So far, the structure coefficients for general multiplayer games have been calculated only for a few population structures, as such calculations often represent a technical challenge [43]. However, once the structure coefficients are known, the containment and volume orders we propose here enable assessment of the consequences of population structure on the evolution of cooperation independently of the game at stake. This way, our approach can help to organize myriads of results on the promotion of cooperation in spatially structured populations.

## 2. Methods and results

### 2.1. Cooperation games and polytopes

We consider symmetric games between *d* players with two strategies, *A* and *B*. A focal player's pay-off depends on the player's own strategy and on the strategies of its *d* – 1 co-players. If *j* co-players play *A*, a focal *A*-player obtains 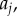 whereas a focal *B*-player obtains 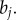 These interactions are represented by the following pay-off table:

**Table:** 

opposing *A*-players	0	1	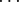	*j*	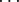	*d* – 1
pay-off to *A*	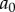	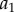	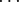	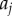	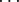	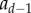
pay-off to *B*	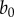	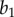	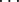	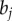	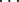	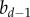

It follows that a game is determined by 2*d* real numbers and can thus be considered as a point in a 2*d*-dimensional space.

In which sense can we say that one population structure favours cooperation more than another? To answer this question precisely, we first need to specify what we mean by ‘cooperation’, as this could refer to different social behaviours, in particular if we move beyond two-player games [44]. We are interested in a particular subset of games that we call ‘cooperation games’. In these games, players decide whether to cooperate (play *A*) or defect (play *B*), and pay-offs are such that: (i) players prefer other group members to cooperate irrespective of their own strategy and (ii) mutual cooperation is favoured over mutual defection. In terms of our pay-off parameters, these conditions imply2.1
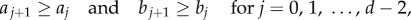
as well as2.2
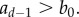


The above conditions are often used to characterize the benefits of cooperation in multiplayer social dilemmas [44], such as the provision of collective goods [19]. However, our conditions do not specify individual costs associated with a decision to cooperate, and hence our class of cooperation games includes not only social dilemmas, but also mutualistic games in which individual and group interests are aligned. If we further restrict pay-offs to values between zero and one,2.3
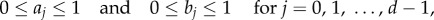
then the set of all cooperation games with *d* players is given by a (convex) polytope [45] in a 2*d*-dimensional space, which we denote by 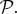 A polytope is a geometric object with flat sides, the generalization of a polygon (which is a two-dimensional polytope) to higher dimensional spaces. See the electronic supplementary material for further details.

We need to specify precisely what we mean by ‘favouring’ cooperation. For our purposes, we say that cooperation is favoured if a single cooperator in a population of defectors has a higher probability of eventually reaching fixation than a single defector in a population of cooperators [46]. This also means that cooperation is more abundant than defection in a mutation-selection process in the limit of low mutation [47]. For weak selection on homogeneous populations of constant size, strategy *A* is favoured over *B* if [42]2.4
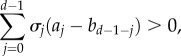
where 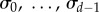 are the *d* structure coefficients. These are independent of pay-offs 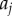 and 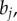 but dependent on the type of spatial structure (for instance, where the co-players of a given focal individual are located) and update rule used to model the evolutionary dynamics. In table 1, we provide examples of population structures and their corresponding structure coefficients (see the electronic supplementary material for a derivation).

**Table 1.** Structure coefficients for some population structures. Parameters *d* and *N* refer to the number of players and population size, respectively. In the group splitting model, *m* is the number of groups and *n* is the group size. The structure coefficients shown here are not normalized; for our purposes it is useful to normalize them so that 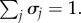

model	structure coefficients	references
Moran process in a well-mixed population	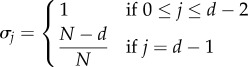	[38]
aspiration dynamics in a well-mixed population	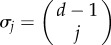	[48]
death–birth process in a cycle ( 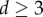 )	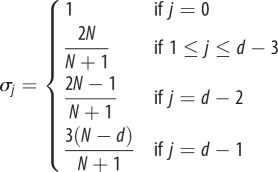	[49]
Moran process in a group splitting model (rare group splitting)	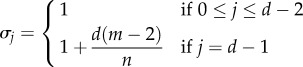	[50]

The structure coefficients are uniquely determined up to a constant factor. Setting one of them to one thus gives a single non-trivial structure coefficient for two-player games [22]. We use the sequence 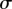 to collect the coefficients and note that, if 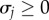 for all *j* and 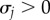 for at least one *j*, we can impose 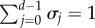 without affecting the selection condition (2.4). For our purposes, this normalization turns out to be more useful than setting one coefficient to one. In particular, such normalization allows us to understand the (normalized) structure coefficients as describing a probability distribution, and to make a straightforward connection with the concept of assortment as developed for the case of linear public goods games [30,51]. To do so, let us rewrite the selection condition (2.4) as2.5
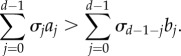


Here, 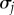 plays the role of the ‘effective’ probability of interacting with *j* individuals of the own type (and *d* – 1 – *j* of the other type). As given by (2.5), the selection condition states that *A* is favoured over *B* if the expected pay-off of an *A*-player is greater than that of a *B*-player when the ‘interaction environments’ [30] are distributed according to 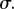


A given population structure will favour cooperation only for a subset of cooperation games. More precisely, for a population structure 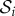 with structure coefficients 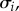 the set of cooperation games for which 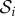 favours *A* over *B* is given by adding the selection condition (2.5) to the inequalities defining the polytope of cooperation games, 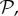 i.e. (2.1)–(2.3). The selection condition (2.5) defines a hyperplane and thus divides the space of games into two: those for which cooperation is favoured and those for which defection is favoured. This shows that our problem is equivalent to a geometric problem in 2*d* dimensions. In the following, we denote by 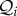 the polytope containing the cooperation games for which cooperation is favoured under population structure 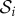 (see the electronic supplementary material).

### 2.2. Containment order

If the set of games 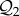 for which cooperation is favoured under population structure 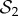 is contained in the set 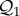 for which cooperation is favoured under population structure 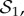 then we say that 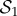 is greater than 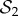 in the containment order [52], and we write 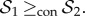 The ordering 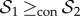 implies that cooperation cannot be favoured under 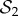 without also being favoured under 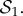


Establishing the containment order is equivalent to a ‘polytope containment problem’ [53], consisting of determining whether or not a polytope is contained in another. Polytope containment problems can be solved numerically by linear programming [54]. Here, we describe an alternative and simpler approach borrowed from the literature on stochastic orders [55]. First, assume that the structure coefficients 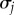 are non-negative and normalized, so that they define a probability distribution over 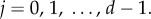 In this case, the left-hand side of the selection condition (2.4) can be interpreted as the expected value 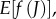 where 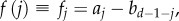 and *J* is the random variable associated with the probability distribution 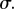 Consider now two population structures 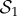 and 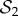 with structure coefficients 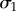 and 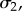 and associated random variables 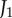 and 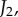 respectively. A sufficient condition leading to the containment order 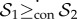 is hence that2.6
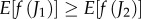
for all cooperation games.

In order to evaluate this condition, we make use of the usual stochastic order [55]. A random variable 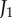 is said to be greater than 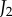 in the stochastic order if and only if 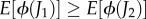 for all increasing functions 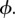 This is denoted by 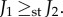 Conveniently, and by (2.1), the sequence 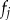 is always increasing in *j*, allowing us to apply this idea directly (see Proposition 1 in the electronic supplementary material for details). One advantage of expressing the containment order in terms of the stochastic order is that we can transform our original polytope containment problem into the problem of finding conditions under which random variables can be stochastically ordered. Some of these conditions follow from a simple inspection of the sequences of structure coefficients. For instance, a sufficient condition leading to the stochastic order 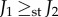 (and hence to the containment order 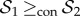) is that 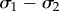 has exactly one sign change from – to + [55]. As we show in §2.4, this simple condition allows us to order different existing models of population structure in a straightforward way.

For the linear public goods game (i.e. a game with pay-offs 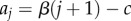 and 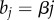 for some 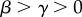 where 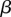 is the marginal benefit from the public good and 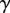 is the individual cost of contributing), the selection condition (2.5) can be put in a form reminiscent of Hamilton's rule with 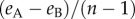 playing the role of a measure of assortment (or relatedness), where 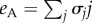 (resp. 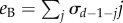) is the mean number of cooperators among the *d* – 1 interaction partners of a cooperator (resp. defector) [51]. For more general cooperation games, the selection condition depends not only on the mean but also on higher moments of the probability distribution given by 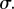 The stochastic order we have used for establishing the containment order is a way of measuring the association between strategies in this general case. Hence, it can be said that population structures greater in the containment order are those characterized by greater ‘effective assortment’ and thus more conducive to the evolution of cooperation. In the extreme case where 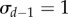 (and 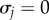 for 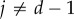), we have the case of a completely segregated population where *A*s only interact with *A*s and *B*s only interact with *B*s. In this case, the selection condition reduces to (2.2), and cooperation is always favoured by definition.

**Figure 2.**
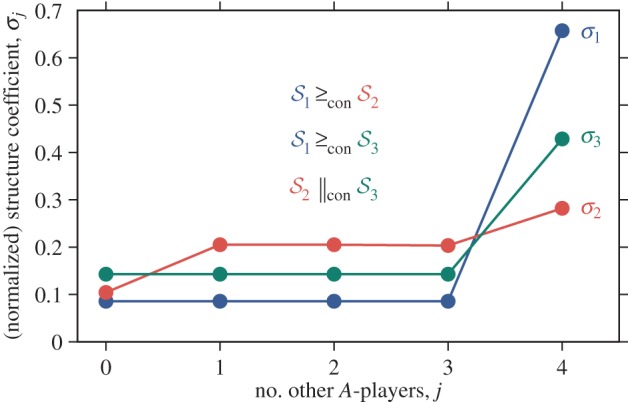
Comparability in the containment order. The structure coefficients 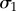 and 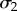 cross exactly once, implying that 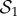 and 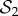 are comparable in the containment order. Moreover, 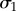 crosses 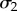 from below; hence 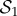 is greater than 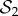 in the containment order 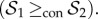 Likewise, 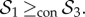 Contrastingly, the structure coefficients 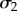 and 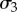 cross exactly twice, implying that 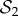 and 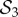 are incomparable in the containment order 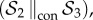 i.e. neither 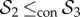 nor 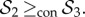 For such cases, the volume order provides an alternative way to order these structures. Here, 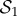 is a group splitting model with *m* = 10 groups of maximum size *n* = 6 and rare probability of splitting (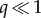), 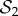 is a cycle of size *N* = 60 and 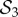 is a group splitting model with *m* = 6, *n* = 10 and 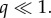

It can happen that neither 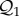 is entirely contained in 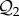 nor 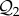 is entirely contained in 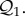 In these cases, 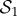 and 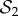 are incomparable in the containment order (i.e. neither 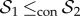 nor 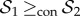 hold) and we write 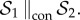 We show in Proposition 2 in the electronic supplementary material that a sufficient condition leading to such incomparability is that the sequences 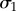 and 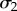 cross twice (figure 2). In this case, there exist both a subset of cooperation games favoured under 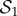 but not under 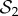 and a subset of cooperation games favoured under 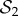 but not under 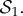

For the commonly discussed case of two-player games in structured populations [22], the sequence 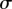 consists of two elements: 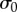 (usually set to one) and 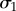 (usually denoted by 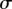 and referred to as ‘the’ structure coefficient). As two sequences of two elements can only cross each other at most once, it follows that any two population structures can be ordered in the containment order if *d* = 2, i.e. the containment order is a total order for two-player games. Moreover, the containment order is given by the comparison of the structure coefficients 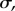 with larger 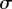 leading to greater containment order. Contrastingly, for 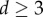 two sequences 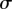 can cross twice. In this case, their respective population structures cannot be compared in the containment order: for multiplayer cooperation games and for the space of all possible population structures, the containment order is only a partial order (see Proposition 3 in the electronic supplementary material).

### 2.3. Volume order

In order to address the cases for which two population structures are incomparable in the containment order, we introduce the ‘volume order’. We say that 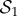 is greater than 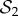 in the volume order, and write 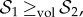 if2.7
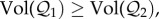
where 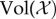 is the volume of polytope 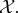 In other words, 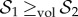 means that for a given *d*, cooperation is favoured under 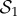 for a greater number of cooperation games than under 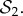 If two structures are ordered in the containment order so that 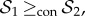 this implies that they are ordered in the volume order so that 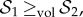 but the converse is not true.

**Figure 3.**
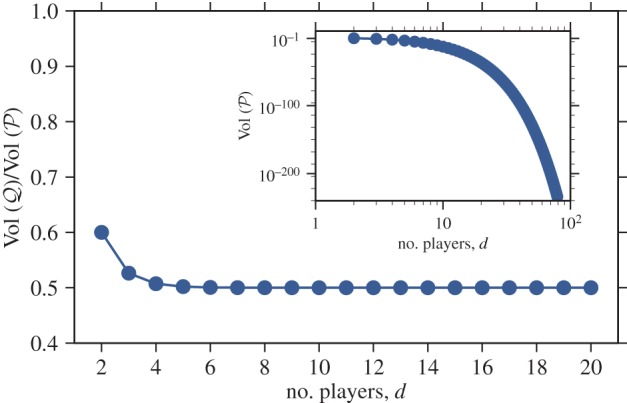
Fraction of cooperation games for which cooperation is favoured in population structures with symmetric structure coefficients (main figure) and volume of cooperation games (inset figure) as functions of the number of players *d*. As *d* increases, the probability that a population structure with symmetric structure coefficients promotes cooperation for a randomly chosen cooperation game quickly approaches 1/2. At the same time, the probability that a randomly chosen game is a cooperation game quickly goes to zero, an effect that seems to be underappreciated in the literature emphasizing the importance of cooperation in evolution.

We find that the volume of all *d*-player cooperation games 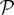 is given by (Proposition 10 in the electronic supplementary material; figure 3):2.8
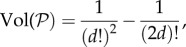
which decreases rapidly with the number of players *d*. For *d* = 2, this volume is equal to 5/24. In this case, the four pay-offs 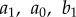 and 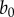 can be ordered in 4! = 24 possible ways, five of which satisfy inequalities (2.1) and (2.2), namely (i) 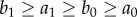 (Prisoner's Dilemma), (ii) 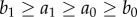 (snowdrift game), (iii) 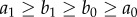 (stag hunt), (iv) 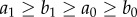 (harmony game) and (v) 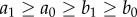 (Prisoner's Delight [56]). For large *d*, condition (2.2) becomes less important and the volume of cooperation games is approximately 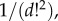 which is the volume of games satisfying conditions (2.1) and (2.3).

For some population structures, such as large well-mixed populations updated with a Moran process, the structure coefficients are symmetric, i.e. 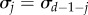 for all *j*. For these cases, the fraction of cooperation games for which cooperation is favoured becomes2.9
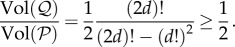


(Proposition 11 in the electronic supplementary material; figure 3). This fraction is equal to 3/5 for *d* = 2, and reduces to 1/2 in the limit of large *d*.

### 2.4. Examples

Let us now illustrate our approach with particular models of spatial structure and associated update rules. Consider first the baseline scenario of a well-mixed population of size 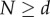 updated with a death–birth (Moran) process [38,46]. In the death–birth process, in each time step, one individual is chosen at random to die and another one is chosen proportional to its pay-off to reproduce by making a copy of itself. We find that for any 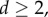 well-mixed populations updated with a death–birth process are ordered in the containment order with respect to the total population size *N*, such that larger populations are more conducive to multiplayer cooperation (Proposition 4 in the electronic supplementary material). Our result generalizes previous results for two-player games and multiplayer games with similar strategies according to which smaller population sizes are less conducive to cooperation because of the stronger local competition among cooperators ([22], eqn 22; [28], eqn B.1). In the limit of large *N* and by equation (2.9), well-mixed populations updated with a death–birth process favour cooperation for exactly one-half of all possible cooperation games.

Consider now the effect of introducing spatial structure while keeping the same update rule. One of the simplest spatial models is the cycle [8]. It has been shown that cycles updated with a death–birth process are better promoters of cooperation than well-mixed populations in the case of two-player games [9,57], and for several examples of multiplayer social dilemmas (such as linear public goods games, snowdrift games and stag hunt games) in the limit of large population size [49]. Our theory allows us to extend these results to all multiplayer cooperation games and arbitrary population sizes. Indeed, we find that cycles are greater than well-mixed populations in the containment order for any given population size *N* (Proposition 6 in the electronic supplementary material). This implies that cycles are better promoters of cooperation than well-mixed populations for any cooperation game, any number of players *d* and any population size *N*.

A second model of spatial structure for which structure coefficients are readily available is the group splitting model of [26]. In this model, a finite population of size *N* is subdivided into *m* groups, which can grow in size and split with probability *q* when reaching the maximum size *n*. In the limit of rare group splitting (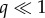), all groups are typically of the maximum size *n* and the structure coefficients can be calculated analytically for general *d*-player games [50]. Consider well-mixed and group splitting populations updated according to a death–birth process. If the number of groups is greater than two, the group splitting model is greater than any well-mixed population in the containment order (Proposition 7 in the electronic supplementary material). Moreover, in the limit of 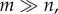 the structure coefficients of the group splitting model become 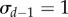 and 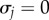 for 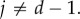 In this limit, the group splitting model is greater in the containment order than any other population structure. Hence, it is the population structure that favours cooperation most among all theoretically possible population structures.

**Figure 4.**
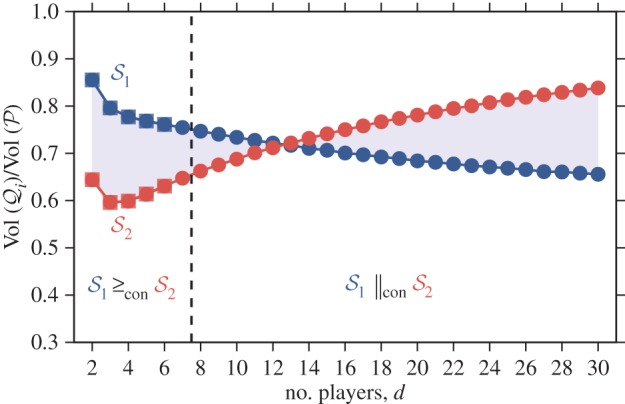
Normalized volumes of cooperation for two different population structures: a cycle of size *N* = 1000 (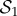) and a group splitting model with *m* = 10 groups of maximum size *n* = 100 (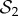). Volumes are calculated exactly for small values of *d* (squares) and approximately using a Monte Carlo method (circles); see appendix A. The cycle is greater than the group splitting model in the volume order for 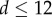 and smaller in this sense for 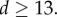 We can also show that the cycle is greater than the group splitting model in the containment order (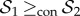) for 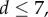 but the two population structures are incomparable in the containment order (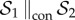) for 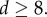

The cycle and the group splitting model are better promoters of cooperation than the well-mixed population. But which one promotes cooperation under more cooperation games, the cycle or the group splitting model? Consider cycles of size *N* and group splitting models with rare group splitting 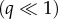 consisting of *m* groups of maximum size *n*, so that the total maximum population size is equal to *N* = *mn*. Assuming that the population size *N* is large, the containment order depends on the number of groups *m* of the group splitting model in the following way (Proposition 8 in the electronic supplementary material). (i) If the number of groups is small 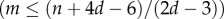 the group splitting model is smaller than the cycle in the containment order. (ii) If the number of groups is intermediate 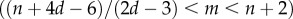 the group splitting model and the cycle are incomparable in the containment order. (iii) If the number of groups is large 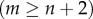 the group splitting model is greater than the cycle in the containment order. As a particular example, consider a cycle of size *N* = 1000 and a group splitting model with *m* = 10 groups of maximum size *n* = 100 (figure 4). In this case, the cycle is greater than the group splitting model in the containment order if 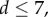 while the two population structures are incomparable in the containment order if 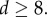 Concerning the volume order, exact computations and numerical simulations suggest that the cycle is greater than the group splitting model for 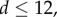 and smaller than the group splitting model otherwise.

Up until now we have compared different models of spatial structure (the well-mixed population, the cycle, the group splitting model) with a single update rule (the Moran death–birth process). However, the structure coefficients depend both on spatial structure and on the update rule. For two-player games, different update rules can have important consequences on the evolutionary dynamics, as they lead to different ‘circles of compensation’, or how far the effects of density dependence extend from a given focal individual [10]. What are the effects of different update rules on multiplayer cooperation games? As an example, consider well-mixed populations with two different update rules: the Moran process, where a random individual dies and its neighbours compete for the empty site, and the aspiration dynamics, where an individual is likely to switch its strategy if the current pay-off does not meet an aspiration level [48,58]. The two update rules can be ordered in the containment order only if 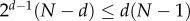 (Proposition 9 in the electronic supplementary material). In this case, aspiration dynamics is greater in the containment order than the Moran process, meaning that if cooperation is favoured under the Moran process it will also be favoured under aspiration dynamics, but not necessarily vice versa. If 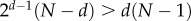 the two structures are incomparable in the containment order. However, for any finite population size *N*, aspiration dynamics is greater in the volume order: overall, cooperation is favoured for more games under aspiration dynamics than under the Moran process.

## 3. Discussion

Our approach to compare models of population structure sheds new light on how to study and analyse the evolution of cooperation in spatially structured populations. We have shown how several existing results, obtained under the assumptions of pairwise interactions, similar strategies or particular classes of multiplayer social dilemmas, generalize to the case of multiplayer cooperation games with distinct strategies that we have considered here. Perhaps more importantly, one can find two population structures such that there is a class of cooperation games for which cooperation is favoured under the first but not under the second, and a class of cooperation games for which the opposite holds true (figure 1*b*). Thus, arbitrarily choosing one or a few games from the set of all possible cooperation games to compare the effects of population structure on the evolution of cooperation can be misleading, even when focusing on the comparison of fixation probabilities under weak selection. This is different from the case of either two-player games or multiplayer games with similar strategies, where a ranking of population structures is always possible in terms of a single real value, and where it is sufficient to focus on a single game without loss of generality [15,22].

We made use of the theory of stochastic orders [55] to provide conditions under which two population structures are comparable or incomparable in the containment order. Within social evolution theory, stochastic orders have recently also been used to tackle the question of whether variability in group size distribution would lead to less stringent conditions for the evolution of cooperation in multiplayer social dilemmas [59]. Our use of stochastic orders in this paper relies on the assumption (fulfilled by all the population structures we used as examples) that the structure coefficients can always be normalized to define a probability distribution. It would be interesting to investigate under which general conditions such an assumption is valid. Another open question is whether two population structures incomparable in the containment order could favour cooperation in disjoint subsets of cooperation games. If the structure coefficients define a probability distribution, this will never be the case, as it will always be possible to find a cooperation game for which the selection condition holds for any two population structures. Consider for instance a game for which 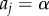 and 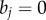 for all *j*, with 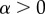 (a mutualistic game where the group optimal action *A* is also individually optimal). In this case, and provided that the structure coefficients are non-negative, the selection condition (2.4) is always satisfied.

We considered a very broad definition of cooperation and a particular measure of evolutionary success, and investigated subset containment relations and volumes of the resulting polytopes. In this respect, our approach is related to a classical study by Mattesi & Jayakar [60], who first defined an ‘altruism domain’ from a set of linear inequalities involving ‘local fitness functions’ and then investigated the problem of finding and measuring the relative volume of the ‘subset of the altruism domain in which *A* is more fit than *B* on average, that is, altruism can evolve’. We note, however, that our definition of cooperation is different from the definition of altruism adopted by Matessi & Jayakar: the ‘multi-level interpretation’ of altruism, in the sense of Kerr *et al*. [44]. In particular, we only focused on the group benefits, not the individual costs, associated with expressing the cooperative action *A*. Such costs could be introduced by adding further sets of inequalities to the ones we used here, for instance by requiring that 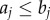 for some or all *j* [19,44]. As we did not specify any costs, our class of cooperation games contains a relatively large set of mutualistic games for which group beneficial behaviours are also individually beneficial. Our measure of evolutionary success is also different, as we focused on the comparison of fixation probabilities in the limit of weak selection, whereas Matessi & Jayakar focused on the one-step change in frequency. Finally, Matessi & Jayakar limited themselves to ‘linear fitness functions’ (equivalent to linear games in our set-up) while we considered more general multiplayer games. The differences between our study and the one by Matessi & Jayakar pinpoint possible future work along these lines. For instance, alternative definitions of cooperation that take into consideration the cost of cooperation [44,61] and exclude mutualistic games could be explored, possibly together with alternative measures of evolutionary success [62]. As long as it is possible to write all conditions as a set of linear inequalities (and hence as polytopes) involving the pay-offs of the game, our definitions can be used and adapted to these cases. It would be interesting to see the extent to which comparisons of different population structures based on the containment and volume orders defined here are robust to changes in the way cooperation and evolutionary success are defined and implemented.

## Supplementary Material

Supplementary Methods: Ordering structured populations in multiplayer cooperation games

## Appendix A. Computing volumes

There are many exact methods for computing volumes of polytopes, including triangulation methods [63] and signed decomposition methods [64]. Computing the exact volume of a polytope is however known to be #P-hard [65], and a simple task only for low dimensions. In figure 4, the volumes for  were calculated exactly using the function volume of the class Polyhedron of the mathematics software Sage (v. 6.5). For  we used a Monte Carlo method for approximating the volumes. For each value of *d*, we randomly generated  increasing sequences  and  and retained only those which fulfilled (2.2). We then checked how many of these sequences verified the selection condition (2.4). The fraction of cooperation games was then approximated by the ratio between these two numbers. Our source code in Python is publicly available on GitHub (https://github.com/jorgeapenas/ordering).

## References

[1] Axelrod R 1984 The evolution of cooperation. New York, NY: Basic Books.

[2] Frank SA 1998 Foundations of social evolution. Princeton, NJ: Princeton University Press.

[3] Hamilton WD 1964 The genetical evolution of social behavior I and II. J. Theoret. Biol. 7, 1–16. (10.1016/0022-5193(64)90038-4) 5875341

[4] Hamilton WD 1964 The genetical evolution of social behavior II. J. Theoret. Biol. 7, 17–52. (10.1016/0022-5193(64)90039-6) 5875340

[5] Nowak MA 2006 Evolutionary dynamics. Cambridge, MA: Harvard University Press.

[6] Allen B, Nowak MA, Dieckmann U 2013 Adaptive dynamics with interaction structure. Am. Nat. 181, E139–E163. (10.1086/670192) 23669549

[7] Débarre F, Hauert C, Doebeli M 2014 Social evolution in structured populations. Nat. Commun. 5, 3409 (10.1038/ncomms4409) 24598979

[8] Ellison G 1993 Learning, local interaction and coordination. Econometrica 61, 1047–1071. (10.2307/2951493)

[9] Grafen A 2007 An inclusive fitness analysis of altruism on a cyclical network. J. Evol. Biol. 20, 2278–2283. (10.1111/j.1420-9101.2007.01413.x) 17956390

[10] Grafen A, Archetti M 2008 Natural selection of altruism in inelastic viscous homogeneous populations. J. Theor. Biol. 252, 694–710. (10.1016/j.jtbi.2008.01.021) 18371985

[11] Hamilton W 1971 Selection of selfish and altruistic behavior in some extreme models. In Man and beast: comparative social behavior (eds Eisenberg JF, Dillon WS), pp. 57–91. Washington, DC: Smithsonian Press.

[12] Ladret V, Lessard S 2007 Fixation probability for a beneficial allele and a mutant strategy in a linear game under weak selection in a finite island model. Theor. Popul. Biol. 72, 409–425. (10.1016/j.tpb.2007.04.001) 17531280

[13] Lehmann L, Keller L, Sumpter DJT 2007 The evolution of helping and harming on graphs: the return of the inclusive fitness effect. J. Evol. Biol. 20, 2284–2295. (10.1111/j.1420-9101.2007.01414.x) 17956391

[14] Lehmann L, Keller L, West S, Roze D 2007 Group selection and kin selection: two concepts but one process. Proc. Natl Acad. Sci. USA 104, 6736–6739. (10.1073/pnas.0700662104) 17416674 PMC1871855

[15] Lehmann L, Rousset F 2010 How life history and demography promote or inhibit the evolution of helping behaviours. Phil. Trans. R. Soc. B 365, 2599–2617. (10.1098/rstb.2010.0138) 20679105 PMC2936172

[16] Nowak MA, Tarnita CE, Antal T 2010 Evolutionary dynamics in structured populations. Phil. Trans. R. Soc. B 365, 19–30. (10.1098/rstb.2009.0215) 20008382 PMC2842709

[17] Ohtsuki H 2010 Evolutionary games in Wright's island model: kin selection meets evolutionary game theory. Evolution 64, 3344–3353. (10.1111/j.1558-5646.2010.01117.x) 20812974

[18] Ohtsuki H, Hauert C, Lieberman E, Nowak MA 2006 A simple rule for the evolution of cooperation on graphs. Nature 441, 502–505. (10.1038/nature04605) 16724065 PMC2430087

[19] Peña J, Nöldeke G, Lehmann L 2015 Evolutionary dynamics of collective action in spatially structured populations. J. Theor. Biol. 382, 122–136. (10.1016/j.jtbi.2015.06.039) 26151588

[20] Rousset F 2004 Genetic structure and selection in subdivided populations. Princeton, NJ: Princeton University Press.

[21] Szabó G, Fáth G 2007 Evolutionary games on graphs. Phys. Rep. 446, 97–216. (10.1016/j.physrep.2007.04.004)

[22] Tarnita CE, Ohtsuki H, Antal T, Fu F, Nowak MA 2009 Strategy selection in structured populations. J. Theor. Biol. 259, 570–581. (10.1016/j.jtbi.2009.03.035) 19358858 PMC2710410

[23] Taylor PD, Day T, Wild G 2007 From inclusive fitness to fixation probability in homogeneous structured populations. J. Theor. Biol. 249, 101–110. (10.1016/j.jtbi.2007.07.006) 17727893

[24] Taylor PD 1992 Altruism in viscous populations—an inclusive fitness approach. Evol. Ecol. 6, 352–356. (10.1007/BF02270971)

[25] Taylor PD, Day T, Wild G 2007 Evolution of cooperation in a finite homogeneous graph. Nature 447, 469–472. (10.1038/nature05784) 17522682

[26] Traulsen A, Nowak MA 2006 Evolution of cooperation by multi-level selection. Proc. Natl Acad. Sci. USA 103, 10 952–10 955. (10.1073/pnas.0602530103) 16829575 PMC1544155

[27] Van Cleve J 2015 Social evolution and genetic interactions in the short and long term. Theor. Popul. Biol. 103, 2–26. (10.1016/j.tpb.2015.05.002) 26003630

[28] Van Cleve J, Lehmann L 2013 Stochastic stability and the evolution of coordination in spatially structured populations. Theor. Popul. Biol. 89, 75–87. (10.1016/j.tpb.2013.08.006) 23999503

[29] Eshel I, Cavalli-Sforza LL 1982 Assortment of encounters and evolution of cooperativeness. Proc. Natl Acad. Sci. USA 79, 1331–1335. (10.1073/pnas.79.4.1331) 16593160 PMC345957

[30] Fletcher JA, Doebeli M 2009 A simple and general explanation for the evolution of altruism. Proc. R. Soc. B 276, 13–19. (10.1098/rspb.2008.0829) PMC2614248 18765343

[31] Platt TG, Bever JD 2009 Kin competition and the evolution of cooperation. Trends Ecol. Evol. 24, 370–377. (10.1016/j.tree.2009.02.009) 19409651 PMC5679087

[32] Gore J, Youk H, van Oudenaarden A 2009 Snowdrift game dynamics and facultative cheating in yeast. Nature 459, 253–256. (10.1038/nature07921) 19349960 PMC2888597

[33] Li X-Y, Pietschke C, Fraune S, Altrock PM, Bosch TCG, Traulsen A 2015 Which games are growing bacterial populations playing? J. R. Soc. Interface 12, 20150121 (10.1098/rsif.2015.0121) 26236827 PMC4528578

[34] Xavier JB, Foster KR 2007 Cooperation and conflict in microbial biofilms. Proc. Natl Acad. Sci. USA 104, 876–881. (10.1073/pnas.0607651104) 17210916 PMC1783407

[35] Hilbe C, Wu B, Traulsen A, Nowak MA 2014 Cooperation and control in multiplayer social dilemmas. Proc. Natl Acad. Sci. USA 111, 16 425–16 430. (10.1073/pnas.1407887111) PMC4246307 25349400

[36] Milinski M, Semmann D, Krambeck H-J, Marotzke M 2006 Stabilizing the Earth's climate is not a losing game: supporting evidence from public goods experiments. Proc. Natl Acad. Sci. USA 103, 3994–3998. (10.1073/pnas.0504902103) 16537474 PMC1449634

[37] Ostrom E 1990 Governing the commons: the evolution of institutions for collective action. Cambridge, UK: Cambridge University Press.

[38] Gokhale CS, Traulsen A 2010 Evolutionary games in the multiverse. Proc. Natl Acad. Sci. USA 107, 5500–5504. (10.1073/pnas.0912214107) 20212124 PMC2851774

[39] Peña J, Lehmann L, Nöldeke G 2014 Gains from switching and evolutionary stability in multi-player matrix games. J. Theor. Biol. 346, 23–33. (10.1016/j.jtbi.2013.12.016) 24380778

[40] Ohtsuki H 2014 Evolutionary dynamics of n-player games played by relatives. Phil. Trans. R. Soc. B 369, 20130359 (10.1098/rstb.2013.0359) 24686931 PMC3982661

[41] Roze D, Rousset F 2008 Multilocus models in the infinite island model of population structure. Theor. Popul. Biol. 73, 529–542. (10.1016/j.tpb.2008.03.002) 18442836

[42] Wu B, Traulsen A, Gokhale CS 2013 Dynamic properties of evolutionary multi-player games in finite populations. Games 4, 182–199. (10.3390/g4020182)

[43] McAvoy A, Hauert C 2015 Structure coefficients and strategy selection in multiplayer games. J. Math. Biol. 72, 203–238. (10.1007/s00285-015-0882-3) 25842359

[44] Kerr B, Godfrey-Smith P, Feldman MW 2004 What is altruism? Trends Ecol. Evol. 19, 135–140. (10.1016/j.tree.2003.10.004) 16701244

[45] Ziegler GM 1995 Lectures on polytopes, vol. 152 Berlin, Germany: Springer.

[46] Nowak MA, Sasaki A, Taylor C, Fudenberg D 2004 Emergence of cooperation and evolutionary stability in finite populations. Nature 428, 646–650. (10.1038/nature02414) 15071593

[47] Fudenberg D, Imhof LA 2006 Imitation processes with small mutations. J. Econ. Theory 131, 251–262. (10.1016/j.jet.2005.04.006)

[48] Du J, Wu B, Altrock PM, Wang L 2014 Aspiration dynamics of multi-player games in finite populations. J. R. Soc. Interface 11, 20140077 (10.1098/rsif.2014.0077) 24598208 PMC3973373

[49] van Veelen M, Nowak MA 2012 Multi-player games on the cycle. J. Theor. Biol. 292, 116–128. (10.1016/j.jtbi.2011.08.031) 21907215 PMC3279760

[50] Kurokawa SS, Ihara Y 2013 Evolution of social behavior in finite populations: a payoff transformation in general n-player games and its implications. Theor. Popul. Biol. 84, 1–8. (10.1016/j.tpb.2012.11.004) 23186609

[51] Bijma P, Aanen DK 2010 Assortment, Hamilton's rule and multilevel selection. Proc. R. Soc. B 277, 673–675. (10.1098/rspb.2009.1093) PMC2842734 19906665

[52] Fishburn PC, Trotter WT 1999 Geometric containment orders: a survey. Order 15, 167–182. (10.1023/A:1006110326269)

[53] Kaibel V, Pfetsch ME 2003 Some algorithmic problems in polytope theory. In Algebra, geometry and software systems (eds Joswig M, Takayama N), pp. 23–47. Berlin, Germany: Springer.

[54] Freund RM, Orlin JB 1985 On the complexity of four polyhedral set containment problems. Math. Program. 33, 139–145. (10.1007/BF01582241)

[55] Shaked M, Shanthikumar JG 2007 Stochastic orders. Berlin, Germany: Springer.

[56] Binmore K 2004 Reciprocity and the social contract. Politics Philos. Econ. 3, 5–35. (10.1177/1470594X04039981)

[57] Ohtsuki H, Nowak MA 2006 Evolutionary games on cycles. Proc. R. Soc. B 273, 2249–2256. (10.1098/rspb.2006.3576) PMC1635521 16901846

[58] Szabó G, Töke C 1998 Evolutionary Prisoner's Dilemma game on a square lattice. Phys. Rev. E 58, 69–73. (10.1103/PhysRevE.58.69)

[59] Peña J, Nöldeke G 2016 Variability in group size and the evolution of collective action. J. Theor. Biol. 389, 72–82. (10.1016/j.jtbi.2015.10.023) 26551151

[60] Matessi C, Jayakar SD 1976 Conditions for the evolution of altruism under Darwinian selection. Theor. Popul. Biol. 9, 360–387. (10.1016/0040-5809(76)90053-8) 785675

[61] Dawes RM 1980 Social dilemmas. Annu. Rev. Psychol. 31, 169–193. (10.1146/annurev.ps.31.020180.001125)

[62] Tarnita CE, Taylor PD 2014 Measures of relative fitness of social behaviors in finite structured population models. Amer. Nat. 184, 477–488. (10.1086/677924) 25226183

[63] Cohen J, Hickey T 1979 Two algorithms for determining volumes of convex polyhedra. J. ACM 26, 401–414. (10.1145/322139.322141)

[64] Lawrence J 1991 Polytope volume computation. Math. Comput. 57, 259–271. (10.1090/S0025-5718-1991-1079024-2)

[65] Dyer M, Frieze A 1988 On the complexity of computing the volume of a polyhedron. SIAM J. Comput. 17, 967–974. (10.1137/0217060)

