# Supplementary material for: Ordering structured populations in multiplayer cooperation games
Source: J R Soc Interface. 2016 Jan;13(114):20150881. doi: 10.1098/rsif.2015.0881 (PMC4759793; doi:10.1098/rsif.2015.0881)
Supplement: Supplementary Methods: Ordering structured populations in multiplayer cooperation games [file rsif20150881supp1.pdf]

Supplementary Material
Supplementary Methods: Ordering structured populations in multiplayer cooperation games

Jorge Peña, Bin Wu, and Arne Traulsen
Department of Evolutionary Theory, Max Planck Institute for Evolutionary Biology,
August-Thienemann-Straße 2, 24306 Plön, Germany,

November 26, 2015

Contents

1 Polytopes **1**

2 Structure coefficients **2**
2.1 Moran process on a well-mixed population 2
2.2 Aspiration dynamics on a well-mixed population 3
2.3 death-Birth process on a cycle 3
2.4 Moran process on a group splitting model 4
2.5 Normalized structure coefficients 6

3 Containment order **7**
3.1 A sufficient condition leading to the containment order 7
3.2 A sufficient condition leading to the incomparability in the containment order 8
3.3 The containment order is a total order for $d = 2$ but a partial order for $d \geq 3$ 10
3.4 Examples 10

4 Volume order **11**

1 Polytopes

A (convex) polyhedron can be defined as the intersection of finitely many closed halfspaces in $\mathbb{R}^n$, i.e., as the set of solutions to a system of m linear inequalities

$$\mathbf{T}\mathbf{x} \leq \mathbf{c}, \tag{1}$$

where $\mathbf{T}$ is a real $m \times n$ matrix, and $\mathbf{c}$ a real vector of size m. A (convex) polytope is a bounded polyhedron. When given by a system of linear inequalities such as (1), a polytope is said to be given in its $\mathcal{H}$-representation (18).

We consider symmetric games with two pure strategies (A and B) between d players. A focal player's payoff depends on its own strategy and on its $d-1$ co-players'. If j co-players play A, a focal player obtains a_j if it plays A or b_j if it plays B. We focus on a subset of games that we call "cooperation games". In these games A represents cooperation; B, defection; and payoffs are such that: (i) irrespective of its own strategy, a focal player prefers co-players to cooperate, and (ii) mutual cooperation is favored over mutual defection. In terms of the payoffs of the game, these conditions respectively imply

$$a_{j+1} \geq a_j \qquad \text{and} \qquad b_{j+1} \geq b_j \qquad \text{for } j = 0, 1, \ldots, d-2, \tag{2}$$

and

$$a_{d-1} > b_0. \tag{3}$$

We further restrict payoffs to values between 0 and 1, so that

$$0 \leq a_j \leq 1 \qquad \text{and} \qquad 0 \leq b_j \leq 1 \qquad \text{for } j = 0, 1, \ldots, d-1. \tag{4}$$

The previous inequalities give the $\mathcal{H}$-representation of a polytope that we denote by $\mathcal{P}$.

For weak selection, strategy A is favored over B if (17)

$$\sum_{j=0}^{d-1} \sigma_j \left(a_j - b_{d-1-j}\right) > 0, \tag{5}$$

where $\sigma_0, \ldots, \sigma_{d-1}$ are the d structure coefficients of the population structure (and associated update rule) under consideration. For a given population structure $\mathcal{S}_i$ with vector of structure coefficients $\boldsymbol{\sigma}_i$, inequalities (2), (3), and (4) together with the selection condition (5) give the $\mathcal{H}$-representation of a polytope that we denote by $\mathcal{Q}_i$.

2 Structure coefficients

The structure coefficients $\boldsymbol{\sigma}$ of a given model of population structure can be calculated from the condition $\rho_A > \rho_B$, where ρ_X denotes the fixation probability of a single mutant playing X (either A or B) in a population of residents playing the opposite strategy (9). This condition can be written in terms of selection coefficients (dependent on the payoffs of the game and the demographic parameters of the model) and expected coalescence times under neutrality (12). However, the expected coalescence times required for calculating the structure coefficients of general d-player games can be difficult to obtain (5, 12). At least for simple population structures, the condition $\rho_A > \rho_B$ can be more easily calculated from first principles, and the structure coefficients extracted from the resulting expressions. This is the approach we followed here.

2.1 Moran process on a well-mixed population

The condition $\rho_A > \rho_B$ is given by (ref. (2), Eq. S19)

$$\sum_{j=0}^{d-1} (Na_j - a_{d-1}) > \sum_{j=0}^{d-1} (Nb_j - b_0),$$

which can be rewritten as

$$\sum_{j=0}^{d-2} N (a_j - b_{d-1-j}) + (N-d)(a_{d-1} - b_0) > 0.$$

Dividing both sides of the inequality by N and comparing with the selection condition (5), we obtain

$$\sigma_j = \begin{cases} 1 & \text{if } 0 \le j \le d-2 \\ \frac{N-d}{N} & \text{if } j = d-1 \end{cases}.$$

2.2 Aspiration dynamics on a well-mixed population

The condition $\rho_A > \rho_B$ is given by (ref. (1), Eq. 3.3)

$$\sum_{j=0}^{d-1} \binom{d-1}{j} (a_j - b_j) > 0.$$

Due to the symmetry of binomial coefficients $\binom{d-1}{j} = \binom{d-1}{d-1-j}$, this can be rewritten as

$$\sum_{j=0}^{d-1} \binom{d-1}{j} (a_j - b_{d-1-j}) > 0.$$

Comparing this last expression with the selection condition (5), we obtain

$$\sigma_j = \binom{d-1}{j}.$$

2.3 death-Birth process on a cycle

Let us consider the model of population structure discussed in ref. (14). Each individual is placed on the node of a cycle. Every sequence of d players defines the participants in a d-player game. Individuals accumulate the payoffs from the d games they are involved in, each with d players. These payoffs are transformed to "fitness" via an exponential payoff-to-fitness mapping (11). Each time step, a randomly chosen individual is selected to die and its two neighbours compete for the vacant spot with a probability proportional to fitness.

If the population starts with a single mutant, mutants form a single connected cluster in the cycle at any time. The state of the population can hence be captured by the number of A-players in this cluster, i. Denote by $f_A^0(i)$ ($f_B^0(i)$) the payoff of an A-player (B-player) lying immediately

at the boundary of a cluster of B-players (A-players), and by $f_A^1(i)$ ($f_B^1(i)$) the payoff of an A-player (B-player) right next to it (Fig. 1). Let $T_i^{\pm}$ be the probability that the number of A-players increases ($+$) or decreases ($-$) in one time step, when there are i A-players in the population. Hence $T_1^- = T_{N-1}^+ = \frac{1}{N}$ and

$$T_i^+ = \frac{2}{N}\frac{\exp(\omega f_A^0(i))}{\exp(\omega f_A^0(i)) + \exp(\omega f_B^1(i))},\ 1 \le i \le N-2, \tag{6a}$$

$$T_i^- = \frac{2}{N}\frac{\exp(\omega f_B^0(i))}{\exp(\omega f_B^0(i)) + \exp(\omega f_A^1(i))},\ 2 \le i \le N-1, \tag{6b}$$

where ω is a parameter measuring the strength of selection. Strategy A is favored if $\rho_A > \rho_B$, which is equivalent to (6)

$$\prod_{i=1}^{N-1} \frac{T_i^+}{T_i^-} > 1. \tag{7}$$

For weak selection, condition (7) is equivalent to

$$\left.\frac{\mathrm{d}}{\mathrm{d}\omega}\prod_{i=1}^{N-1} \frac{T_i^+}{T_i^-}\right|_{\omega=0} > 0. \tag{8}$$

Replacing (6) into (8), we get

$$\begin{aligned}\left.\frac{\mathrm{d}}{\mathrm{d}\omega}\prod_{i=1}^{N-1} \frac{T_i^+}{T_i^-}\right|_{\omega=0} &= \left.\sum_{i=1}^{N-1}\frac{\mathrm{d}}{\mathrm{d}\omega}\frac{T_i^+}{T_i^-}\right|_{\omega=0} \\ &= \left.\sum_{i=1}^{N-1} N\left(\frac{\mathrm{d}}{\mathrm{d}\omega}T_i^+ - \frac{\mathrm{d}}{\mathrm{d}\omega}T_i^-\right)\right|_{\omega=0} \\ &= \frac{1}{2}\sum_{i=1}^{N-1} f_A^0(i) + \frac{1}{2}\sum_{i=2}^{N-1} f_A^1(i) - \frac{1}{2}\sum_{i=1}^{N-1} f_B^0(i) - \frac{1}{2}\sum_{i=1}^{N-2} f_B^1(i).\end{aligned} \tag{9}$$

The above expression is a linear combination of the payoff entries a_j and b_j. Further, by the selection condition (5), the coefficients of a_j are the same as those of b_{d-1-j}. Thus it is only necessary to calculate the coefficients of a_j, which only depend on the first two terms of (9). Using the expressions for $f_A^0(i)$ and $f_A^1(i)$ (see ref. (14), Appendix B) we finally obtain:

$$\sigma_j = \begin{cases} 1 & \text{if } j = 0 \\ \frac{2N}{N+1} & \text{if } 1 \le j \le d-3 \\ \frac{2N-1}{N+1} & \text{if } j = d-2 \\ \frac{3(N-d)}{N+1} & \text{if } j = d-1 \end{cases}. \tag{10}$$

It is noteworthy that the expression

$$(a_0 - b_0) + 2\sum_{i=1}^{d-2}(a_i - b_i) + 3(a_{d-1} - b_{d-1}) > 0,$$

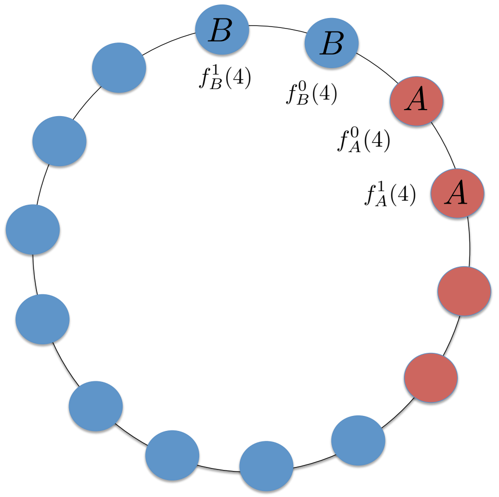

Figure 1: Example of the death-birth process on a cycle. The population is embedded on a cycle of size $N = 14$. There are $i = 4$ A-players. $f_A^0(i)$ (resp. $f_B^0(i)$) is the payoff of an A-player (resp. B-player) next to the boundary between A-players and B-players. $f_A^1(i)$ (resp. $f_B^1(i)$) is the payoff of an A-player (resp. B-player) second-to-next to the boundary between A-players and B-players.

obtained in ref. (14) is a sufficient condition for strategy A to be more abundant than strategy B. Our result (5) with structure coefficients given by (10) is the necessary and sufficient condition. In addition, we note that our result holds for general payoff-to-fitness mapping f, provided that $f'(0)$ is non-vanishing (16). Finally, we note that the stricture coefficients given in (10) are valid for $d \geq 3$. For $d = 2$, they are given by $\sigma_0 = 1$ and $\sigma_1 = (3N - 8)/N$ (see Eq. 38, Ref. (9)).

2.4 Moran process on a group splitting model

Consider the following multiplayer extension (4) of the group splitting model of ref. (10). The population is subdivided into m groups. Each population is allowed to grow to its maximum size n, then splits with probability q. Within populations, random groups of d individuals form and interact in a d-player game. When group splitting is rare ($q \ll 1$) and the mapping between payoffs and fitness is given by an exponential function, the ratio of fixation probabilities is given by (ref. (4), Eq. 15):

$$\frac{\rho_A}{\rho_B} = \exp\left[\frac{m+n-2}{d} w \sum_{j=0}^{d-1} (C_j + \kappa B_j)\right], \tag{11}$$

where

$$C_j = a_j - b_j,$$
$$B_j = j\,(a_j - a_{j-1}) + (d-1-j)\,(b_{j+1} - b_j)\,,$$

are, respectively, the "direct" and "indirect" gains from switching from strategy A to strategy B (see ref. (7), Eqs. 6 and 7), and

$$\kappa = \frac{m-2}{m+n-2} \tag{12}$$

can be interpreted as the "scaled relatedness coefficient" of this model when the migration rate is zero (see ref. (13), Eq. B.4).

From (11), a necessary and sufficient condition for $\rho_A > \rho_B$ is that:

$$\sum_{j=0}^{d-1} (C_j + \kappa B_j) > 0, \tag{13}$$

which can be rearranged in terms of payoffs and structure coefficients in the form of the left hand side of (5):

$$\sum_{j=0}^{d-1} (C_j + \kappa B_j) = \sum_{j=0}^{d-1} a_j - \sum_{j=0}^{d-1} b_j + \kappa \left[d a_{d-1} - \sum_{j=0}^{d-1} a_j - d b_0 + \sum_{j=0}^{d-1} b_j \right].$$

Hence, condition (13) can be written as:

$$(1-\kappa) \sum_{j=0}^{d-2} (a_j - b_{d-1-j}) + (1 - \kappa + \kappa d)(a_{d-1} - b_0) > 0.$$

After dividing both sides of the inequality by $1-\kappa$, inserting the value of κ given in (12), and comparing with (5), we obtain

$$\sigma_j = \begin{cases} 1 & \text{if } 0 \leq j \leq d-2 \\ 1 + \frac{d(m-2)}{n} & \text{if } j = d-1 \end{cases}.$$

2.5 Normalized structure coefficients

For all population structures discussed in this section and listed in Table 1 of the main text, the structure coefficients are nonnegative. This is also true for many other population structures, at least for $d = 2$ (9). In these cases, the structure coefficients can be normalized so that the containment order can be investigated using stochastic orders (8). Henceforth, we refer to the normalized structure coefficients by $\boldsymbol{\sigma} = (\sigma_0, \ldots, \sigma_{d-1})$. Table 1 lists the normalized structure coefficients for the examples of population structures previously discussed.

3 Containment order

3.1 A sufficient condition leading to the containment order

Consider two population structures $\mathcal{S}_1$ and $\mathcal{S}_2$ characterized by the (normalized) structure coefficients $\boldsymbol{\sigma}_1$ and $\boldsymbol{\sigma}_2$, and associated random variables J_1 and J_2, respectively. Let us also define the

Model	Normalized structure coefficients
Moran process on a well-mixed population	$\sigma_j = \begin{cases} \frac{N}{d(N-1)} & \text{if } 0 \leq j \leq d-2 \\ \frac{N-d}{d(N-1)} & \text{if } j = d-1 \end{cases}$
Aspiration dynamics on a well-mixed population	$\sigma_j = \frac{\binom{d-1}{j}}{2^{d-1}}$
death-Birth process on a cycle ($d \geq 3$)	$\sigma_j = \begin{cases} \frac{N+1}{d(2N-3)} & \text{if } j = 0 \\ \frac{2N}{d(2N-3)} & \text{if } 1 \leq j \leq d-3 \\ \frac{2N-1}{d(2N-3)} & \text{if } j = d-2 \\ \frac{3(N-d)}{d(2N-3)} & \text{if } j = d-1 \end{cases}$
Moran process on a group splitting model	$\sigma_j = \begin{cases} \frac{n}{d(m+n-2)} & \text{if } 0 \leq j \leq d-2 \\ \frac{n+d(m-2)}{d(m+n-2)} & \text{if } j = d-1 \end{cases}$

Table 1: Normalized structure coefficients for multiplayer evolutionary game models.

sequence of "gains from flipping":

$$f(j) \equiv f_j = a_j - b_{d-1-j}, \tag{14}$$

i.e., the gains in payoff experienced by a focal B-player interacting with j A-players (and $d-1-j$ B-players) after all players in the group, including the focal, flip their strategies (A-players become B-players and vice versa).

Because of condition (2), the gains from flipping are increasing. Hence, a sufficient condition for $\mathcal{S}_1$ to be greater than $\mathcal{S}_2$ in the containment order is that

$$\mathrm{E}\left[\phi(J_2)\right] \geq 0 \Rightarrow \mathrm{E}\left[\phi(J_1)\right] \geq 0, \text{ for all increasing functions } \phi : \mathbb{R} \to \mathbb{R}. \tag{15}$$

A sufficient condition for this is that

$$\mathrm{E}\left[\phi(J_1)\right] \geq \mathrm{E}\left[\phi(J_2)\right], \text{ for all increasing functions } \phi : \mathbb{R} \to \mathbb{R}, \tag{16}$$

which is fulfilled by definition if J_1 is greater than J_2 in the (usual) stochastic order, denoted by $J_1 \geq_{\mathrm{st}} J_2$ (ref. (8), p. 4).

There are many conditions leading to the stochastic ordering of two random variables (ref. (8), ch. 1). For instance, it is known that $J_1 \geq_{\mathrm{st}} J_2$ if and only if (ref. (8), p. 4)

$$\varsigma_{1,k} \leq \varsigma_{2,k} \text{ for all } k = 0, 1, \ldots, d-1, \tag{17}$$

where ς is the distribution function corresponding to $\boldsymbol{\sigma}$, i.e.,

$$\varsigma_k = \Pr(J \leq k) = \sum_{j=0}^{k} \sigma_j, \text{ for } k = 0, 1, \ldots, d-1. \tag{18}$$

A simple sufficient condition leading to the set of inequalities given by (17) and hence to $J_1 \geq_{\mathrm{st}} J_2$ is that $S^-(\boldsymbol{\sigma}_1 - \boldsymbol{\sigma}_2) = 1$ (where $S^-(a)$ is the number of sign changes of the sequence a) and

the sign sequence is $-,+$ (ref. (8), p. 10). That is, if the structure coefficients $\boldsymbol{\sigma}_1$ “put more weight” in larger values of j than the structure coefficients $\boldsymbol{\sigma}_2$, then $J_1 \geq_{\mathrm{st}} J_2$. We summarize this observation in the following proposition.

Proposition 1 (A sufficient condition leading to the containment order)**.** *Let $\mathcal{S}_1$ and $\mathcal{S}_2$ be two population structures with (normalized) structure coefficients $\boldsymbol{\sigma}_1$ and $\boldsymbol{\sigma}_2$, respectively. If $S^-(\boldsymbol{\sigma}_1 - \boldsymbol{\sigma}_2) = 1$ and the sign sequence is $-,+$, then $\mathcal{S}_1 \geq_{\mathrm{con}} \mathcal{S}_2$.*

3.2 A sufficient condition leading to the incomparability in the containment order

Given two population structures $\mathcal{S}_1$ and $\mathcal{S}_2$, it could be that neither $\mathcal{S}_1 \geq_{\mathrm{con}} \mathcal{S}_2$ nor $\mathcal{S}_1 \leq_{\mathrm{con}} \mathcal{S}_2$ holds true. We are also interested in establishing a simple sufficient condition leading to such incomparability in the containment order, that we denote by $\mathcal{S}_1 \parallel_{\mathrm{con}} \mathcal{S}_2$. In order to derive this, suppose that the structure coefficients of $\mathcal{S}_1$ and $\mathcal{S}_2$ cross each other twice, i.e., that

$$S^-(\boldsymbol{\sigma}_1 - \boldsymbol{\sigma}_2) = 2. \tag{19}$$

Condition (19) implies that $S^-(\varsigma_1 - \varsigma_2) = 1$ (ref. (15), p. 621) and hence that (17) does not hold true. This in turn implies that J_1 and J_2 are incomparable in the stochastic order, i.e., $J_1 \parallel_{\mathrm{st}} J_2$. Showing that (19) also implies $\mathcal{S}_1 \parallel_{\mathrm{con}} \mathcal{S}_2$ however requires some additional arguments. Indeed, note that $J_1 \parallel_{\mathrm{st}} J_2$ does not necessarily imply $\mathcal{S}_1 \parallel_{\mathrm{con}} \mathcal{S}_2$: the stochastic order is a sufficient but not a necessary condition leading to the containment order (cf. (15) and (16)).

In order to prove that (19) leads to $\mathcal{S}_1 \parallel_{\mathrm{con}} \mathcal{S}_2$, we make use of two other stochastic orders: the increasing convex order and the increasing concave order (ref. (8), p. 181). A random variable J_1 is said to be greater than J_2 in the increasing convex (resp. concave) order, denoted by $J_1 \geq_{\mathrm{icx}} J_2$ (resp. $J_1 \geq_{\mathrm{icv}} J_2$), if

$$\mathrm{E}\left[\phi(J_1)\right] \geq \mathrm{E}\left[\phi(J_2)\right], \text{ for all increasing convex (resp. concave) functions } \phi : \mathbb{R} \to \mathbb{R}. \tag{20}$$

A simple condition leading to these orders is given in the following lemma.

Lemma 1 (A sufficient condition leading to the increasing convex (resp. concave) order)**.** *Let X and Y be two random variables with density functions p and q respectively. If*

$$S^-(p - q) = 2 \text{ with sign sequence } +,-,+ \text{ (resp. } -,+,-) \tag{21}$$

then $X \geq_{\mathrm{icx}} Y$ (resp. $X \geq_{\mathrm{icv}} Y$).

Proof. Denote by P and Q the distribution functions associated to X and Y, respectively. Condition (21) implies (ref. (15), p. 621)

$$S^-(P - Q) = 1 \text{ with sign sequence } +,- \text{ (resp. } -,+). \tag{22}$$

Since (22) implies the increasing convex (resp. concave) order (ref. (8), p. 194), this completes the proof. □

Let us now consider two population structures $\mathcal{S}_1$ and $\mathcal{S}_2$ whose structure coefficients satisfy (19). Without loss of generality, suppose that the sign pattern is $+, -, +$. By Lemma 1, it follows that $J_1 \geq_{\text{icx}} J_2$ and $J_2 \geq_{\text{icv}} J_1$. This suggests that there might be both (i) games with increasing and convex gains from flipping f_j for which $\mathcal{S}_1$ (but not $\mathcal{S}_2$) fulfills the selection condition (5), and (ii) games with increasing and concave gains from flipping f_j for which $\mathcal{S}_2$ (but not $\mathcal{S}_1$) fulfills the selection condition (5).

As an example of such games, consider a club goods game between cooperators (A) and defectors (B), where cooperators pay a cost $c > 0$ in order to provide an excludable collective good that only cooperators can use, while defectors refrain from contributing and hence from using the good (7). This game is characterized by the payoff sequences:

$$a_j = v_{j+1},$$
$$b_j = c,$$

where v_k gives the value of the collective good as a function of the total number of cooperators, $k = j + 1$, and c is the payoff defectors obtain. We further assume that v_k is given by

$$v_k = v \sum_{\ell=0}^{k-1} u^\ell = v \frac{1 - u^k}{1 - u},$$

where $v > 0$ is some baseline value, and $u > 0$ is a synergy or discounting parameter (3). Furthermore, we require that $v/c > \gamma$, where $1/\gamma = (1 - u^d)/(1 - u)$, so that (3) is fulfilled.

The gains from flipping of this game are then given by

$$f_j = v_{j+1} - c.$$

Let us first impose the condition:

$$\mathrm{E}\left[f(J_1)\right] > 0 > \mathrm{E}\left[f(J_2)\right] \tag{23}$$

so that $\mathcal{S}_1$ but not $\mathcal{S}_2$ satisfies the selection condition (5). Condition (23) is satisfied if

$$\frac{1}{\gamma_1} < \frac{v}{c} < \frac{1}{\gamma_2} \tag{24}$$

where

$$\gamma_i = \mathrm{E}\left[\frac{1 - u^{J_i+1}}{1 - u}\right], \text{ for } i = 1, 2.$$

Note that (24) is satisfied if $u > 1$, because in this case $(1 - u^{j+1})/(1 - u)$ is increasing and convex and $J_1 \geq_{\text{icx}} J_2$. Additionally, $1/\gamma \leq 1/\gamma_1$ always holds.

Let us now impose the condition:

$$\mathrm{E}\left[f(J_2)\right] > 0 > \mathrm{E}\left[f(J_1)\right] \tag{25}$$

so that $\mathcal{S}_2$ but not $\mathcal{S}_1$ satisfies the selection condition (5). In this case, (25) is satisfied if

$$\frac{1}{\gamma_2} < \frac{v}{c} < \frac{1}{\gamma_1}, \tag{26}$$

which holds true if $0 < u < 1$, as in this case $(1 - u^{j+1})/(1 - u)$ is increasing and concave and $J_2 \geq_{\mathrm{icv}} J_1$. Additionally, $1/\gamma \leq 1/\gamma_2$ always holds.

We summarize the previous observations in the following proposition.

Proposition 2 (A sufficient condition leading to the incomparability in the containment order)**.** *Let $\mathcal{S}_1$ and $\mathcal{S}_2$ be two population structures with (normalized) structure coefficients $\boldsymbol{\sigma}_1$ and $\boldsymbol{\sigma}_2$, respectively. If $S^-(\boldsymbol{\sigma}_1 - \boldsymbol{\sigma}_2) = 2$ then $\mathcal{S}_1 \parallel_{\mathrm{con}} \mathcal{S}_2$, i.e., neither $\mathcal{S}_1 \geq_{\mathrm{con}} \mathcal{S}_2$ nor $\mathcal{S}_1 \leq_{\mathrm{con}} \mathcal{S}_2$ hold true. Moreover, if the sign sequence of $\boldsymbol{\sigma}_1 - \boldsymbol{\sigma}_2$ is $+, -, +$ (resp. $-, +, -$) then it is possible to find cooperation games with convex (resp. concave) gains from flipping f_j such that the selection condition* (5) *is satisfied for $\mathcal{S}_1$ but not for $\mathcal{S}_2$ (resp. for $\mathcal{S}_2$ but not for $\mathcal{S}_1$) and cooperation games with concave (resp. convex) gains from flipping f_j such that the selection condition* (5) *is satisfied for $\mathcal{S}_2$ but not for $\mathcal{S}_1$ (resp. for $\mathcal{S}_1$ but not for $\mathcal{S}_2$).*

3.3 The containment order is a total order for $d = 2$ but a partial order for $d \geq 3$

Propositions 1 and 2 allow us to prove the following result.

Proposition 3 (The containment order is total for $d = 2$ but partial for $d = 3$)**.** *Consider the set of all possible population structures $\{\mathcal{S}\}$ for a given group size d. $\{\mathcal{S}\}$ is totally ordered under $\leq_{\mathrm{con}}$ for $d = 2$ but only partially ordered under $\leq_{\mathrm{con}}$ for $d \geq 3$.*

Proof. For $d = 2$, the probability mass function given by the normalized structure coefficients $\boldsymbol{\sigma}$ consists of only two points. Consequently, $\boldsymbol{\sigma}_1 - \boldsymbol{\sigma}_2$ has either (i) no sign changes (i.e., $\boldsymbol{\sigma}_1 = \boldsymbol{\sigma}_2$), which implies $\mathcal{S}_1 =_{\mathrm{con}} \mathcal{S}_2$; (ii) a sign change from $-$ to $+$, which implies $\mathcal{S}_1 \geq_{\mathrm{con}} \mathcal{S}_2$; or (iii) a sign change from $+$ to $-$, which implies $\mathcal{S}_1 \leq_{\mathrm{con}} \mathcal{S}_2$. For $d \geq 3$, the probability mass function given by the normalized structure coefficients $\boldsymbol{\sigma}$ consists of $d \geq 3$ points. In this case, it is always possible to find $\mathcal{S}_1$ and $\mathcal{S}_2$ such that $\boldsymbol{\sigma}_1 - \boldsymbol{\sigma}_2$ has two sign changes. In this case, neither $\mathcal{S}_1 \geq_{\mathrm{con}} \mathcal{S}_2$ nor $\mathcal{S}_1 \leq_{\mathrm{con}} \mathcal{S}_2$ hold true. □

3.4 Examples

In the following, we state several results concerning the containment order for the population structures listed in Table 1. We omit the proofs, as they are straightforward applications of Propositions 1 and 2 above.

Proposition 4 (Containment order for well-mixed populations updated with a Moran process). *Denote by $\mathcal{S}_N^{\text{well-mixed}}$ a well-mixed population of size N with a Moran process as updating rule. Then $\mathcal{S}_N^{\text{well-mixed}} \leq_{\text{con}} \mathcal{S}_{N+1}^{\text{well-mixed}}$ for all $N \geq d$.*

Proposition 5 (Containment order for cycles updated with a Moran death-Birth process). *Denote by $\mathcal{S}_N^{\text{cycle}}$ a cycle of size N with death-Birth updating. Then $\mathcal{S}_N^{\text{cycle}} \leq_{\text{con}} \mathcal{S}_{N+1}^{\text{cycle}}$ for all $N \geq d$.*

Proposition 6 (Containment order for cycles and well-mixed populations updated with a Moran death-Birth process). *Let $\mathcal{S}_N^{\text{cycle}}$ and $\mathcal{S}_N^{\text{well-mixed}}$ be respectively a cycle and a well-mixed population of size N, both updated with a Moran death-Birth process. Then, for all d and all $N > d$, $\mathcal{S}_N^{\text{cycle}} \geq_{\text{con}} \mathcal{S}_N^{\text{well-mixed}}$.*

Proposition 7 (Containment order for group splitting models and well-mixed populations, both updated with a Moran death-Birth process). *Let $\mathcal{S}_{m,n}^{\text{group-splitting}}$ be a group splitting model with m groups of maximum size n and rare group splitting ($q \ll 1$), and $\mathcal{S}_N^{\text{well-mixed}}$ a well-mixed population of size N, both updated with a Moran death-Birth process. We have that $\mathcal{S}_{1,N}^{\text{group-splitting}} =_{\text{con}} \mathcal{S}_N^{\text{well-mixed}}$, $\mathcal{S}_{2,n}^{\text{group-splitting}} =_{\text{con}} \mathcal{S}_{\infty}^{\text{well-mixed}}$ for any n, and $\mathcal{S}_{m,n}^{\text{group-splitting}} \geq_{\text{con}} \mathcal{S}_{\infty}^{\text{well-mixed}}$ for $m \geq 3$ and any n.*

Proposition 8 (Containment order for cycles and group splitting models, both updated with a Moran death-Birth process). *Let $\mathcal{S}_{m,n}^{\text{group-splitting}}$ be a group splitting model with m groups of maximum size n and rare group splitting ($q \ll 1$), and $\mathcal{S}^{\text{cycle}}$ be a cycle of size N, both updated with a Moran death-Birth process. In the limit of large $N = mn$ we have:*

1. *If $m \leq \frac{n+4d-6}{2d-3}$ then $\mathcal{S}_{m,n}^{\text{group-splitting}} \leq_{\text{con}} \mathcal{S}_N^{\text{cycle}}$.*
2. *If $\frac{n+4d-6}{2d-3} < m < n+2$ then $\mathcal{S}_{m,n}^{\text{group-splitting}} \parallel_{\text{con}} \mathcal{S}_N^{\text{cycle}}$.*
3. *If $m \geq n+2$, then $\mathcal{S}_{m,n}^{\text{group-splitting}} \geq_{\text{con}} \mathcal{S}_N^{\text{cycle}}$.*

Proposition 9 (Aspiration dynamics vs. Moran process in well-mixed populations). *Let $\mathcal{S}^{\text{aspiration}}$ and $\mathcal{S}_N^{\text{Moran}}$ be well-mixed populations of size $N \geq d$, updated with aspiration dynamics and a Moran process, respectively. We have:*

1. *If $2^{d-1}(N-d) \leq d(N-1)$ then $\mathcal{S}_N^{\text{Moran}} \leq_{\text{con}} \mathcal{S}^{\text{aspiration}}$.*
2. *If $2^{d-1}(N-d) > d(N-1)$ then $\mathcal{S}_N^{\text{Moran}} \parallel_{\text{con}} \mathcal{S}^{\text{aspiration}}$.*

4 Volume order

In the following, we give a formula for the volume of cooperation games, as defined by inequalities (2)–(4). For this, we find convenient to define $\mathcal{I}$ as the polytope given by inequalities (2) and (4), and $\mathcal{J}$ as the polytope given by inequalities (2), (4), and $a_{d-1} \leq b_0$ (which is the opposite of (3)). The volumes of these two polytopes are easy to calculate exactly using probabilistic arguments. Indeed, we have the following two lemmas.

Lemma 2 (Volume of $\mathcal{I}$). *We have that*

$$\textit{Vol}(\mathcal{I}) = \frac{1}{(d!)^2}.$$

Proof. Calculating the volume of $\mathcal{I}$ is equivalent to calculating the probability that two sequences $(a_0, \ldots, a_{d-1})$ and $(b_0, \ldots, b_{d-1})$, with elements randomly and independently drawn from the interval $[0, 1]$, are such that $a_0 \leq a_1 \leq \ldots \leq a_{d-1}$ and $b_0 \leq b_1 \leq \ldots \leq b_{d-1}$. For each sequence, the probability of having a randomly ordered sequence of length d is $1/d!$, since $d!$ is the number of permutations of d distinct objects and only one of such permutations will be given in the specified order. Since the two sequences are independent, the total probability is given by $1/(d!)^2$. □

Lemma 3 (Volume of $\mathcal{J}$). *We have that*

$$\textit{Vol}(\mathcal{J}) = \frac{1}{(2d)!}.$$

Proof. Calculating the volume of $\mathcal{J}$ is equivalent to calculating the probability that the sequence

$$(a_0, \ldots, a_{d-1}, b_0, \ldots, b_{d-1})$$

with elements randomly and independently drawn from the interval $[0, 1]$ is such that $a_0 \leq a_1 \leq \ldots \leq a_{d-1} \leq b_0 \leq b_1 \leq \ldots \leq b_{d-1}$. Following the same argument as in the proof of Lemma 2, this probability is equal to $1/(2d)!$. □

Making use of these two lemmas, we can find an expression for the volume of $\mathcal{P}$, the polytope of cooperation games. We state this result in the following proposition.

Proposition 10 (Volume of cooperation games). *The volume of cooperation games is given by*

$$\textit{Vol}(\mathcal{P}) = \frac{1}{(d!)^2} - \frac{1}{(2d)!}$$

Proof. Follows from Lemmas 2 and 3 upon noticing that $\mathcal{P} = \mathcal{I} - \mathcal{J}$. □

Some population structures, such as large well-mixed populations updated with a Moran process, and finite well-mixed populations updated with the aspiration dynamics, are such that their structure coefficients are symmetric, i.e., $\sigma_j = \sigma_{d-1-j}$ for all j. We are intereted in calculating the fraction of cooperation games for which such population structures favor strategy A. In order to calculate this result, we need the following lemma. The lemma may appear to be obvious for symmetry reasons, but the additional requirement that we are dealing with cooperation games (and hence a subset of the hypercube of all possible games) adds a further complication.

Lemma 4. *Let $\mathcal{S}$ be a population structure with symmetric structure coefficients, i.e., $\sigma_j = \sigma_{d-1-j}$ for all j. Let also $\mathcal{I}_+$ be the subset of $\mathcal{I}$ for which A is favored over B. Then*

$$\frac{\mathrm{Vol}(\mathcal{I}_+)}{\mathrm{Vol}(\mathcal{I})} = \frac{1}{2}.$$

Proof. Denote by $\mathcal{I}_-$ and $\mathcal{I}_0$ the subsets of $\mathcal{I}$ such that $\sum_{j=0}^{d-1}\sigma_j(a_j-b_{d-1-j})<0$ and $\sum_{j=0}^{d-1}\sigma_j(a_j-b_{d-1-j})=0$, respectively. Then we arrive at a partition of set $\mathcal{I}$, namely $\mathcal{I}=\mathcal{I}_+\cup\mathcal{I}_0\cup\mathcal{I}_-$. Since there must exist a j^* such that $\sigma_{j^*}\neq 0$, the solution space of $\sum_{j=0}^{d-1}\sigma_j(a_j-b_{d-1-j})=0$ is of lower dimension than $2d$, and hence $\mathrm{Vol}(\mathcal{I}_0)=0$. Thus $\mathrm{Vol}(\mathcal{I})=\mathrm{Vol}(\mathcal{I}_+)+\mathrm{Vol}(\mathcal{I}_-)$.

In the following, we prove that $\mathrm{Vol}(\mathcal{I}_+)=\mathrm{Vol}(\mathcal{I}_-)$. For this, we define the mapping

$$\psi((a_0,a_1,\cdots,a_{d-1},b_0,b_1,\cdots,b_{d-1}))=(b_0,b_1,\cdots,b_{d-1},a_0,a_1,\cdots,a_{d-1}). \tag{27}$$

For every $(a_0,a_1,\cdots,a_{d-1},b_0,b_1,\cdots,b_{d-1})\in\mathcal{I}_+$, $\sum_{j=0}^{d-1}\sigma_j(a_j-b_{d-1-j})>0$ holds, which implies that $\sum_{j=0}^{d-1}\sigma_j(b_{d-1-j}-a_j)<0$ holds. Since $\sigma_j=\sigma_{d-1-j}$, we have $\sum_{j=0}^{d-1}\sigma_{d-1-j}(b_{d-1-j}-a_j)=\sum_{j=0}^{d-1}\sigma_j(b_j-a_{d-1-j})<0$. This implies that $\psi((a_0,a_1,\cdots,a_{d-1},b_0,b_1,\cdots,b_{d-1}))\in\mathcal{I}_-$. Thus $\psi(\mathcal{I}_+)\subset\mathcal{I}_-$. Similarly, we have $\psi(\mathcal{I}_-)\subset\mathcal{I}_+$. Therefore $\psi(\mathcal{I}_+)=\mathcal{I}_-$. This leads to

$$\mathrm{Vol}(\mathcal{I}_-)=\left|\int_{\mathcal{I}_-}dx\right|=\left|\int_{\psi(\mathcal{I}_+)}dx\right|. \tag{28}$$

In addition, since ψ^2 is the identity mapping ($\psi^2=I$), ψ is invertible and the inverse mapping is the mapping itself, i.e., $\psi^{-1}=\psi$. This leads to

$$\mathrm{Vol}(\mathcal{I}_-)=\left|\int_{\mathcal{I}_+}d\psi^{-1}(x)\right|=\left|\int_{\mathcal{I}_+}d\psi(x)\right|=\left|\int_{\mathcal{I}_+}\det(\psi)dx\right|. \tag{29}$$

Here $\det(\psi)$ is the determinant of the Jacobian matrix of the transformation ψ at x. Further, considering that ψ is a linear mapping, $\psi^2=I$ implies $|\det(\psi)|=1$. Thus (29) yields

$$\mathrm{Vol}(\mathcal{I}_-)=\left|\int_{\mathcal{I}_+}dx\right|=\mathrm{Vol}(\mathcal{I}_+). \tag{30}$$

Therefore $\mathrm{Vol}(\mathcal{I})=\mathrm{Vol}(\mathcal{I}_+)+\mathrm{Vol}(\mathcal{I}_-)=2\mathrm{Vol}(\mathcal{I}_+)$, or

$$\frac{\mathrm{Vol}(\mathcal{I}_+)}{\mathrm{Vol}(\mathcal{I})}=\frac{1}{2}.$$

□

With this lemma, we can prove the following proposition.

Proposition 11. *Let $\mathcal{S}$ be a population structure with positive symmetric structure coefficients, i.e., $\sigma_j=\sigma_{d-1-j}$ for all j, and $\mathcal{Q}$ the polytope asociated to all cooperation games for which A is favored over B under $\mathcal{S}$. Then*

$$\frac{\mathrm{Vol}(\mathcal{Q})}{\mathrm{Vol}(\mathcal{P})}=\frac{1}{2}\frac{(2d)!}{(2d)!-(d!)^2},$$

which is a decreasing function of d, and is equal to $1/2$ in the limit of large d.

Proof. It is easy to check that for every $(a_0,a_1,\cdots,a_{d-1},b_0,b_1,\cdots,b_{d-1})\in\mathcal{J}$, $\sum_{j=0}^{d-1-j}\sigma_j(a_j-b_{d-1-j})\leq(a_{d-1}-b_0)\sum_{j=0}^{d-1-j}\sigma_j=(a_{d-1}-b_0)<0$ holds true, which implies $\mathcal{J}\subset\mathcal{I}_-$. Since

$\mathcal{J} \subset \mathcal{I}_-$ and $\mathcal{I} = \mathcal{I}_- \cup \mathcal{I}_0 \cup \mathcal{I}_+$, then $\mathcal{Q} = \mathcal{I}_+$. Moreover $\mathcal{P} = \mathcal{I} - \mathcal{J}$. Hence

$$\frac{\mathrm{Vol}(\mathcal{Q})}{\mathrm{Vol}(\mathcal{P})} = \frac{\mathrm{Vol}(\mathcal{I}_+)}{\mathrm{Vol}(\mathcal{I}) - \mathrm{Vol}(\mathcal{J})},$$

and by Lemma 4

$$\frac{\mathrm{Vol}(\mathcal{Q})}{\mathrm{Vol}(\mathcal{P})} = \frac{1}{2}\frac{\mathrm{Vol}(\mathcal{I})}{\mathrm{Vol}(\mathcal{I}) - \mathrm{Vol}(\mathcal{J})}.$$

Using Lemmas 2 and 3, we finish the proof. □

References

[1] J. Du, B. Wu, P. M. Altrock, and L. Wang. Aspiration dynamics of multi-player games in finite populations. *Journal of Royal Society Interface*, 11(94):1742–5662, 2014.

[2] C. S. Gokhale and A. Traulsen. Evolutionary games in the multiverse. *Proceedings of the National Academy of Sciences USA*, 107:5500–5504, 2010.

[3] C. Hauert, F. Michor, M. A. Nowak, and M. Doebeli. Synergy and discounting of cooperation in social dilemmas. *Journal of Theoretical Biology*, 239:195–202, 2006.

[4] S. S. Kurokawa and Y. Ihara. Evolution of social behavior in finite populations: A payoff transformation in general n-player games and its implications. *Theoretical Population Biology*, 84, March 2013.

[5] V. Ladret and S. Lessard. Fixation probability for a beneficial allele and a mutant strategy in a linear game under weak selection in a finite island model. *Theoretical Population Biology*, 72(3):409–425, 2007.

[6] M. A. Nowak, A. Sasaki, C. Taylor, and D. Fudenberg. Emergence of cooperation and evolutionary stability in finite populations. *Nature*, 428:646–650, 2004.

[7] J. Peña, G. Nöldeke, and L. Lehmann. Evolutionary dynamics of collective action in spatially structured populations. *Journal of Theoretical Biology*, 382:122–136, 2015.

[8] M. Shaked and J. G. Shanthikumar. *Stochastic orders*. Springer, 2007.

[9] C. E. Tarnita, H. Ohtsuki, T. Antal, F. Fu, and M. A. Nowak. Strategy selection in structured populations. *Journal of Theoretical Biology*, 259:570–581, 2009.

[10] A. Traulsen and M. A. Nowak. Evolution of cooperation by multi-level selection. *Proceedings of the National Academy of Sciences USA*, 103:10952–10955, 2006.

[11] A. Traulsen, N. Shoresh, and M. A. Nowak. Analytical results for individual and group selection of any intensity. *Bulletin of Mathematical Biology*, 70:1410–1424, 2008.

[12] J. Van Cleve. Social evolution and genetic interactions in the short and long term. *Theoretical Population Biology*, 103:2–26, 2015.

[13] J. Van Cleve and L. Lehmann. Stochastic stability and the evolution of coordination in spatially structured populations. *Theoretical Population Biology*, 89(0):75–87, 2013.

[14] M. van Veelen and M. A. Nowak. Multi-player games on the cycle. *Journal of Theoretical Biology*, 292:116–128, 2012.

[15] W. Whitt. Uniform conditional variability ordering of probability distributions. *Journal of Applied Probability*, 22(3):619–633, 1985.

[16] B. Wu, P. M. Altrock, L. Wang, and A. Traulsen. Universality of weak selection. *Physical Review E*, 82:046106, 2010.

[17] B. Wu, A. Traulsen, and C. S. Gokhale. Dynamic properties of evolutionary multi-player games in finite populations. *Games*, 4(2):182–199, 2013.

[18] G. M. Ziegler. *Lectures on polytopes*, volume 152. Springer, 1995.
